# Pharmacokinetics, pharmacodynamics, and safety of prandial oral insulin (N11005) in healthy subjects

**DOI:** 10.3389/fendo.2023.1172327

**Published:** 2023-07-26

**Authors:** Qi Pan, Xiaoxia Wang, Wenjia Li, Xiaofeng Chen, Yulei Zhuang, Qinghong Zhou, Yuhui Huang, Yijie Zhou, Li Lan, Zhijie Wang, Wenjia Wang, Juan Hong, Wei-Hua Hao, Yu-Tsai Yang, Lixin Guo

**Affiliations:** ^1^ Department of Endocrinology, Beijing Hospital, National Center of Gerontology, Institute of Geriatric Medicine, Chinese Academy of Medical Sciences, Beijing, China; ^2^ Sunshine Lake Pharma Co., Ltd., Dongguan, China; ^3^ INNOPHARMAX, INC., Taipei, Taiwan

**Keywords:** oral insulin, pharmacokinetics, pharmacodynamics, safety, healthy subjects

## Abstract

**Aims:**

To verify whether the oral insulin N11005 is administered as a prandial insulin by assessing the pharmacokinetics (PK), pharmacodynamics (PD), and safety profiles of N11005 with a short-acting biosynthetic human insulin (Novolin R) as reference.

**Methods:**

This was a randomized, open-label, single-dose, crossover hyperinsulinemic-euglycemic clamp study in healthy Chinese male subjects. A total of 12 subjects were enrolled in the test (T) group (N11005, 300 IU, p.o.) and the reference (R) group (Novolin R, 0.1 IU/Kg, i.h.) with a washout period of 14 days. All subjects were administered on the same day of the clamp study. Glucose Infusion Rates (GIR), serum insulin, and C-peptide concentration were determined during every 8-hour clamp cycle. Trial registration: Clinicaltrials.gov identifier NCT04975022.

**Results:**

After administration, the ratios of mean serum C-peptide concentration to baseline concentration in both T and R groups were lower than 50%, which confirmed the stability of the clamp platform. T group (N11005) showed a more rapid onset of action (tGIR_10%max_≈11 min) and a comparable duration of action to the R group, which was basically in line with the characteristics of prandial insulins. No adverse events (AEs) occurred throughout the study, which demonstrated that N11005 and Novolin R are safe and well-tolerated.

**Conclusions:**

The PD profiles of the single-dose N11005 in the human body are similar to those of prandial insulins, with an excellent safety profile.

**Clinical trial registration:**

Clinicaltrials.gov, identifier NCT04975022.

## Introduction

Diabetes is caused by a relative or absolute deficiency in insulin secretion in the patient’s body. There are two forms of normal insulin secretion: continuous secretion at basal state, i.e., basal insulin secretion, and secretion in response to exogenous stimuli (mainly meal-stimulated response), i.e., prandial insulin secretion. Abnormalities in the early phase of insulin secretion are early signs of insulin secretion defects in the progress of Type 2 diabetes mellitus (T2DM) and exist throughout the course of diabetes, which constitute the main cause of postprandial hyperglycemia that is an independent risk factor for all-cause death and fatal cardiovascular death (1–3). Postprandial glucose control is beneficial in maintaining hemoglobin A1c (HbA1c) levels within the normal range and controlling risk factors of cardiovascular diseases in patients with diabetes mellitus. Therefore, it is of great significance to control postprandial glucose with prandial insulin supplements. Guidelines for Management of Postmeal Glucose in Diabetes (4) released by the International Diabetes Federation (IDF) and Chinese Expert Consensus on Management of Postprandial Hyperglycemia for Type 2 Diabetes Mellitus (5) recommend that short-acting human insulins and rapid-acting insulin analogs are used to supplement the inadequate prandial insulins to control post-meal hyperglycemia. Currently, most commercially available prandial insulins, including Novolin R, NovoRapid, and Humalog, are administered *via* injection, which causes pain and inconvenience to patients and reduces compliance with treatment. In addition, long-term injection activities will cause adverse reactions at injection sites. For this reason, it is urgent to develop a new easy-to-use, effective, safe, and reliable insulin formulation.

N11005 is an oral insulin with human insulin as its active ingredient. Drawing on studies related to insulin molecular structure, characteristics of solid self-microemulsifying preparations, and oral absorption of insulin, N11005 was developed and modified as a rapid-acting oral dosage form using Oralpas Pro^®^, a new solid self-microemulsifying system. After oral administration, it will rapidly disperse due to gastric peristalsis into uniform-sized microemulsion droplets by which insulin molecules are wrapped to prevent degradation by gastric acid, enzymes, and intestinal fluid, hence improving the stability of the drug in the digestive tract. To investigate the permeability of N11005, we performed the *in vitro* study using the Caco-2 cell model. When N11005 or the control group (free insulin) were incubated with Caco-2 cells for 30 min, the cumulative amount transported was 6.53% and the Papp was 16*10^-6^cm/s for N11005 while no insulin was detected in the control group, illustrating that the formulation of N11005 can promote insulin transmembrane absorption. To investigate gastrointestine absorption and rapid-action of N11005, we performed mechanistic studies in Rats. Prevention of intestinal absorption by pyloric ligation, followed by intragastric N11005 dosing resulted in insulin plasma concentrations in 15 min was comparable to those observed in unligated rats received oral dosing, illustrating that absorption of oral N11005 can rapidly occur in the stomach. Preclinical PD studies in animals also demonstrated that N11005 had hypoglycemic effects after single administration in normal rats and dogs, the T_max_ values were 7.5-15min and 15min, respectively, and the insulin level returned to baseline in 2 hours.

In this study, we analyzed the PK, PD, and safety profiles of N11005 in healthy subjects by using the hyperinsulinemic-euglycemic clamp technique.

## Methods

This is a randomized, open-label, crossover study under fasting and hyperinsulinemic-euglycemic clamp conditions in healthy Chinese subjects, which was conducted at Beijing Hospital (the clinical study site) with the approval of its Drug Clinical Trial Institutional Ethics Committee in accordance with the Declaration of Helsinki, Good Clinical Practice (GCP), and applicable laws and regulations. All subjects involved have signed informed consent forms (ICFs).

The objective of this study was to preliminarily verify whether oral insulin N11005 is a prandial insulin by assessing the PK, PD, and safety profiles of N11005. A total of 12 healthy male subjects aged 18 to 45 years were enrolled in a test group (oral insulin N11005, 300IU, p.o.; T group) and a reference group (reference drug Novolin R, 0.1 IU/Kg, i.h.; R group). This study was designed to include a 10-day screening period (Day -14 ~ -4), a 2-day preparation period (Day -3~ -1), and a 2-day treatment period: from the admission of subjects to the Clinical Site on the day prior to drug administration per cycle (Day -1) to the end of that clamp cycle (2 days in total). The next cycle of administration started after a washout period of 14 days (during which relevant examinations were performed, such as physical examinations, vital signs measurements, 12-lead electrocardiogram (ECG), hematology, blood biochemistry, and urinalysis), and then subjects entered the 7-days follow-up period.

For the duration of the study, blood glucose levels were measured every 5 min within 2 h prior to and 0~4 h after drug administration, and subsequently measured every 10 min within 4~8 h after drug administration. The glucose infusion rates (GIR) were calculated. At the same time, blood samples were obtained for the measurement of insulin and C-peptide levels at the following time points: every 30 min within 2 h before administration and 5, 10, 20, 30, 40, 50, 60, 75, 90, 105, 120, 150, 180, 210, 240, 270, 300, 330, 360, 420, and 480 min post drug administration.

### Rationale for dosage determination

According to dosing requirements in Technical Guidelines for Research, Development, and Evaluation of Biosimilars (Trial) (6)issued by the Center for Drug Evaluation (CDE), National Medical Products Administration (NMPA), “Comparative studies on PK and PD variability of drugs shall be designed on the basis of the most sensitive population, parameters, dosage, route of administration and testing methods, and scientific demonstration is needed for required sample size determination. The study shall apply the administration route and dose of the reference drug or a sensitive dose under which the difference is more likely to be shown.” and “Comparative PK studies shall be conducted in the most sensitive population where the difference is most easily detected and with the dose in the steepest part of the dose-effect curve, which can usually be investigated in PK/PD studies”. The human dose range converted from the effective animal dose ED30 was estimated to be 300-833 IU, with the adult body weight calculated at 60 kg. In addition, the data from animal toxicology trials showed that the no observable adverse effect level (NOAEL) of dogs was determined to be 100 IU/kg from which a corresponding Human Equivalent Dose (HED) was extrapolated to be 50 IU/kg using body surface area (BSA) normalization method, and 3000 IU based on an adult body weight of 60 kg. Considering the differences in pharmacological activity and PK profiles between animals and humans, the limitations of animal models, the receptor characteristics, and other factors, the first-in-human (FIH) dose was initially adjusted to be 300 IU by dividing an appropriate factor of safety (determined to be 10 in this study). The relative bioavailability of N11005 administered by subcutaneous injection in dogs was 1.83% (0.89%~4.44%). The oral dose of N11005 was set at 300 IU (T group), and its corresponding subcutaneous dose was 5.49 IU. Considering the Instructions for Use (IFU) of Novolin R, relevant regulations and requirements, clinical safety of drug administration, and feasibility of sample testing, the reference group (R group) in this study was administered at a dose of 0.1 IU per kg of body weight.

### Hyperinsulinemic-euglycemic clamps

All subjects were admitted to the study site one day prior to the start of the clamp test and fasted overnight after receiving a standard meal before 8:00 p.m. On the morning of the test day, with all subjects in fasting condition and supine position, intravenous blood collection and infusion channels were established by means of an indwelling catheter inserted *via* retrograde venipuncture of the forearm superficial veins and infused with normal saline to maintain channel patency to facilitate blood sampling (PK/PD blood samples were collected through this channel). A heated box was used to obtain arterialized venous blood by keeping the temperature around that arm at about 50~60°C, and then an intravenous catheter was inserted into the median cubital vein for insulin and glucose infusion.

At the start of the test, short-acting human insulin (human insulin) was injected using a syringe pump through the median cubital vein at a rate of 2.0 mU/kg/min for the first 10 minutes, and 1.0 mU/kg/min for the next 590 minutes simultaneously with 20% glucose solution being infused.

Plasma glucose levels were measured for blood samples obtained every 10 minutes through the forearm’s superficial venous access. The infusion rate of glucose, 20% (w/v), was adjusted, based on measured blood glucose levels, to maintain blood glucose at ±10% of the target value. The target blood glucose value during this clamp test was equal to the baseline glucose value subtracting 0.28 mmol/L (5 mg/dL), of which the baseline glucose value was the average of three plasma glucose values measured by intravenous blood sampling at 10-minute intervals after the subject arm was placed in the heated box for 15 minutes.

After confirming that GIR was stable, investigators gave either the study drug (oral insulin N11005, 300 IU, p.o.) or the reference drug (Novolin R, 0.1 IU/kg, i.h. in the abdomen).

### PK

Serum insulin and C-peptide concentrations at 2 hours before and 8 hours after drug administration were analyzed using a validated enzyme-linked immunosorbent assay (ELISA). PK parameters were analyzed *via* noncompartmental analysis techniques using Phoenix WinNonlin^®^ (Version 8.1). The peak concentration (C_max_) and time to C_max_ (T_max_) were presented as measured values. The elimination half-life (t_1/2_) of serum insulin was calculated by area under the curve (AUC). The AUC for insulin from time zero to 2 h, 4 h, 8 h, and infinite time after dosing was calculated using the trapezoidal rule.

### PD

The PD profiles were assessed by GIR in this study. The area under the curve of GIR (AUC_GIR_) was calculated *via* the trapezoidal method using the Phoenix WinNonlin^®^ (Version 8.1) software according to GIR at 2 h, 4 h, and 8 h after drug administration. The maximum GIR (GIR_max_) and the time to GIR_max_ (tGIR_max_) were presented as measured values. The time to early 10% of GIR_max_ (early tGIR_10%max_) and early 50% of GIR_max_ (early tGIR_50%max_) were calculated based on measured values.

### Safety assessments

For the duration of the study, all subjects were monitored in terms of adverse events (AEs), vital signs, laboratory examinations, electrocardiogram indicators, and other safety parameters.

### Statistical analyses

All statistical analyses were performed using SAS 9.4 programming (SAS Institute Inc., North Carolina, USA). PK and PD parameters were descriptively expressed as N, arithmetic mean, standard deviation (SD), coefficient of variation (CV), median, minimum, and maximum. Primary PK and PD parameters were analyzed using multivariate analysis of variance (ANOVA) after logarithmic conversion. The 90% confidence interval (CI) was used to evaluate the relative bioavailability of the drugs, with intraindividual CV (intra-CV%) calculated. The mixed-effect model was adopted in ANOVA, with treatment, period, and sequence of administration as fixed effects, and subjects (sequence of administration) as random effects. The relative bioavailability of the study drug was calculated by the ratio of the AUC value of the normalized dose of oral insulin to that under injection, as follows:

Bioavailability = (AUC_oral_/AUC_injection_)* (Dose_injection_/Dose_oral_) *100

Data were analyzed using nonparametric tests considering the small sample size of this study. The two-side test was used to determine the significances between pairs of measurements. Differences with *P*<0.05 was considered statistically significant while those with *P>*0.05 was considered not statistically significant.

## Results

### Demographics and other baseline characteristics

A total of 12 healthy male adult subjects were enrolled in this study. Subjects were considered as TR sequence and RT sequence based on the sequence of their drug administration (study drug: oral insulin, 300 IU. p.o. or subcutaneous injection of reference drug). The mean age (SD) in the two sequences was 29.8 (5.19) and 27.8 (2.50) respectively; mean body weight (kg, SD) was 62.98 (7.067) and 66.40 (4.428) respectively; and mean BMI (kg/m^2^, SD) was 22.045 (1.8659) and 21.753 (1.4750) respectively. Both sequences of patients had no previous medical history, surgical history, or combined medication. The demographic baseline data of the two treatment groups were similar; their overall baseline characteristics and demographic data met the eligibility criteria as specified in the protocol. All subjects completed study medications. The baseline blood glucose values of all subjects were within the normal range, indicating good compliance during the study.

### Quality assessments of clamp test

**Figure 1:**
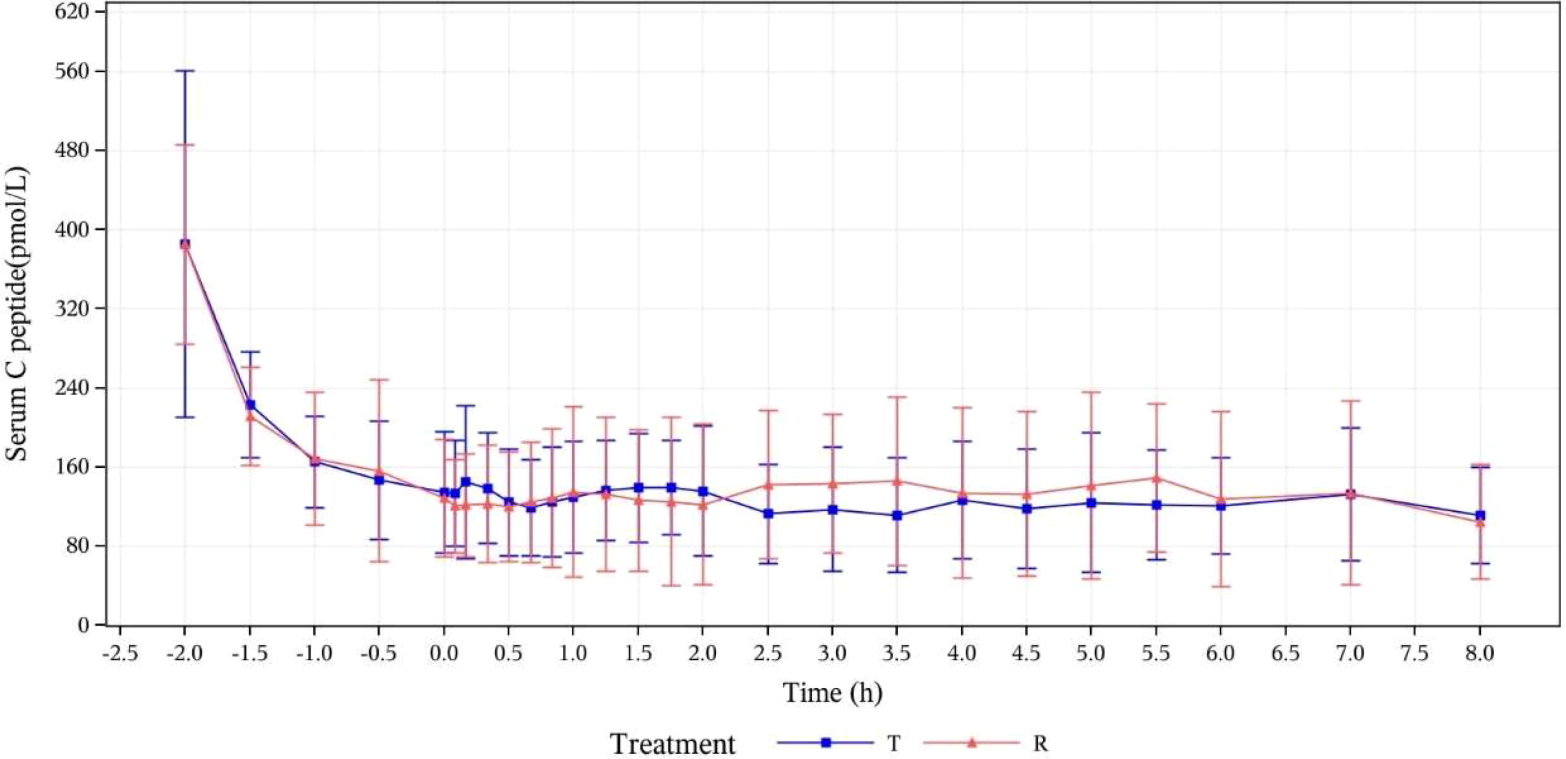
Mean concentration-time curves for serum C-peptide (pmol/L) at specified time points (PK concentration set). T, study drug, 300 IU, p.o.; R, reference drug, 0.1 IU/Kg, i.h.

**Figure 2:**
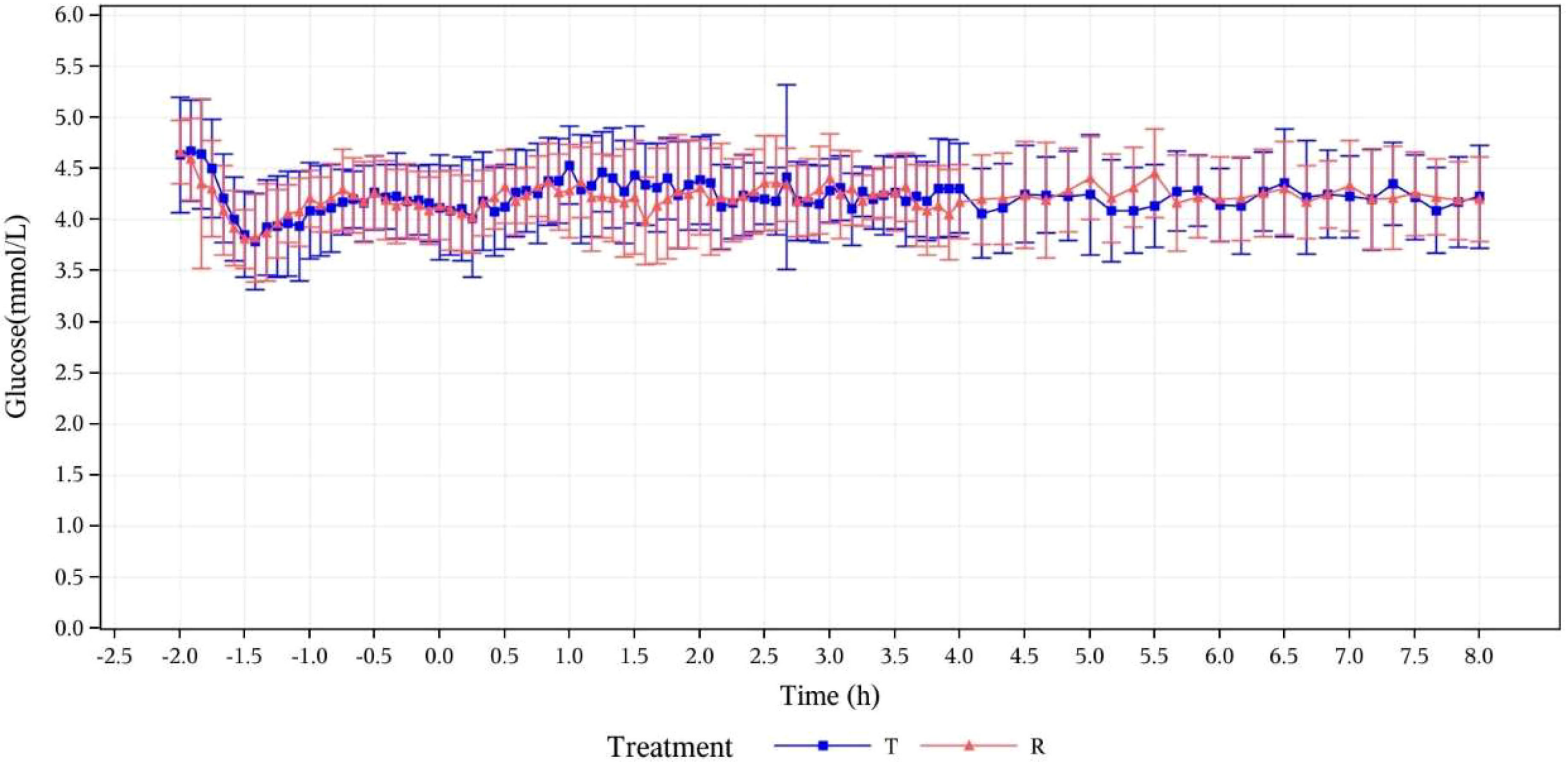
Concentration-time curves for serum glucose (mmol/L) at specified time points. T, study drug, 300 IU, p.o.; R, reference drug, 0.1 IU/Kg, i.h.

The baseline serum C-peptide level was defined as the arithmetic mean of serum C-peptide concentrations measured at -120, -90, -60, -30, and 0 min before drug administration. The mean of serum C-peptide levels and the changes from baseline in T group and R group were respectively calculated at the following time points: 5, 10, 20, 30, 40, 50, 60, 75, 90, 105, 150, 120, 180, 210, 240, 270, 300, 330, 360, 420, and 480 min after drug administration. After administration, the means of serum C-peptide levels (mean ± SD) of subjects in the T group and R group were 126.77 ± 49.619 and 130.17 ± 67.753, respectively. The ratios of the mean serum C-peptide value to baseline in the two groups were 32.6% and 33.2%, respectively. After the subjects were in a stable state, C peptide levels in both groups were lower than 50% of the baseline levels, which verified that the dominant insulin concentration achieved by exogenous intravenous infusion had inhibited the production of endogenous insulin in subjects. The euglycemic clamp platform was stable (**Figure 1**). During the clamp tests, measured blood glucose values were within the range of ± 10% of the target level (baseline blood glucose average-0.28 mmol/L), as shown in **Figure 2**.

### PK assessments

#### Serum insulin concentration

**Figure 3:**
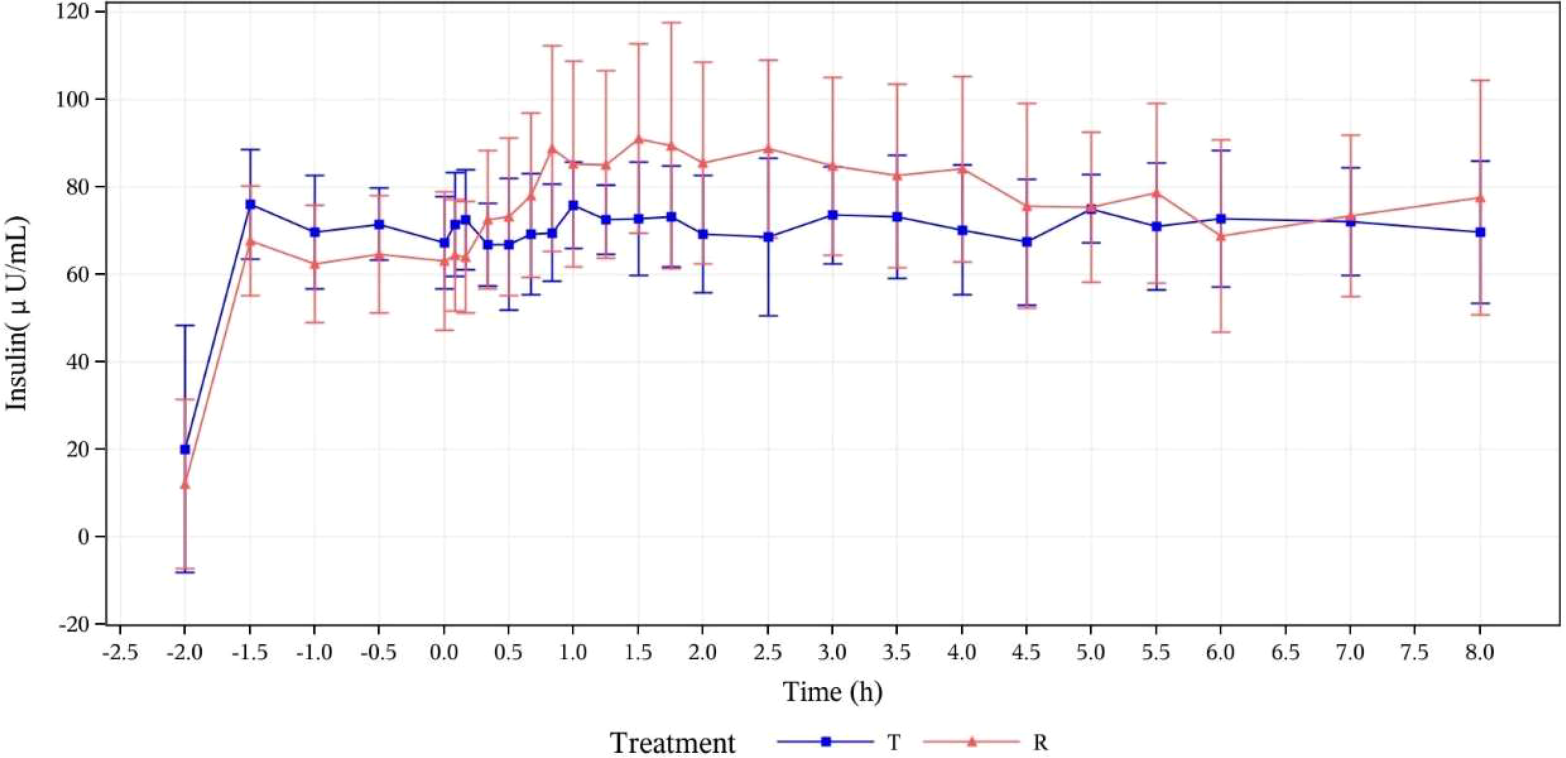
Mean serum insulin concentration (μU/mL) versus time curves (PK analysis set). T, study drug, 300 IU, p.o.; R, reference drug, 0.1 IU/Kg, i.h.

Serum insulin concentration levels were detected in all subjects after they received the test or reference drugs. The two treatment groups showed basically comparable serum insulin concentration levels and trends of concentration change, as demonstrated by mean serum insulin concentration (μU/mL) versus time curves in **Figure 3**.

#### PK parameters of serum insulin

PK parameters of oral insulin (T group) and Novolin R (R group) are presented in **Table 1**. PK parameters of two groups were analyzed based on the PKP analysis set: C_max_ mean ± SD (CV%) was 16.33 ± 5.227 μU/mL (32.02%) vs. 40.30 ± 14.552 μU/mL (36.11%); T_max_ median (minimum, maximum) was 4.980 (0.07~7.97) h vs. 1.730 (0.8~7.98) h; t_1/2_ mean ± SD was 2.985 ± 2.5691 h vs. 2.825 ± 3.0532 in T group and R group, respectively. The relative bioavailability of serum insulin was calculated as follows:

**Table 1:** Summary of PK parameters (PK parameter set).

PK parameters (unit)	T Group	R Group
	(mean ± sd)	(mean ± sd)
C_max_ (μU/mL)	16.33 ± 5.227	40.30 ± 14.552
AUC_0-2h_ (h*μU/mL)	8.808 ± 6.3928	37.538 ± 16.1951
AUC_0-4 h_ (h*μU/mL)	18.417 ± 12.7240	81.220 ± 27.8741
AUC_0-8 h_ (h*μU/mL)	37.142 ± 25.5412	133.280 ± 53.1209
AUC_0-∞_ (h*μU/mL)	52.572 ± 40.9234	198.500 ± 201.5001
T_max_ (h)^a^	4.980 (0.07,7.97)	1.730 (0.8,7.98)
t_1/2_ (h)	2.985 ± 2.5691	2.825 ± 3.0532
F (%)	0.710 ± 0.5107	

a:T_max_ is presented as median (minimum, maximum).

T group: study drug, 300 IU, p.o.; R group: reference drug, 0.1 IU/Kg, i.h.

F= (AUC_0-8 h, oral_/AUC_0-8 h, injection_) * (Dose_injection_/Dose_oral_) *100%

The relative bioavailability of serum insulin (F%) in subjects from T group was calculated to be (0.710 ± 0.5107) %.

### PD assessments

#### GIR

**Figure 4:**
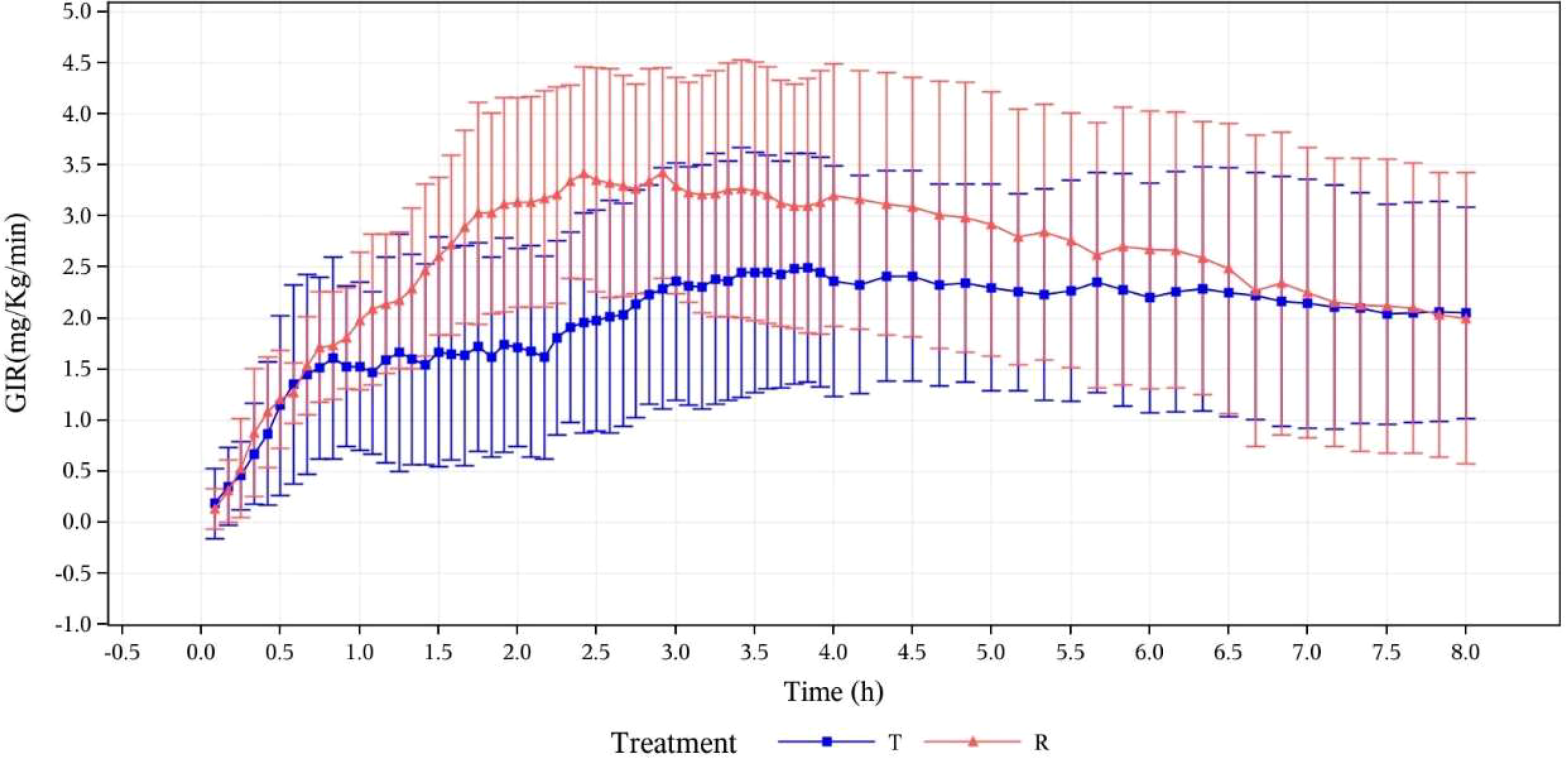
Mean GIR (mg/kg/min) calculated after baseline correction versus time curves (pharmacodynamic analysis set). T, study drug, 300 IU, p.o.; R, reference drug, 0.1 IU/Kg, i.h.

In this study, the PD concentration analysis of 12 subjects was based on the PDC analysis set. The blood glucose-lowering properties of the study drug (oral insulin) were reflected by the change of GIR after drug administration. The GIR values of the two groups were observed to be gradually increasing after oral administration of oral insulin and subcutaneous injection of Novolin R. The GIR variations indicated that the study and reference drugs were all absorbed. More details are shown by mean GIRs ( ± SD) calculated after baseline correction versus time curves in **Figure 4**.

#### GIR parameters

GIR parameters of oral insulin (T) and Group Novolin R (R) are presented in

**Table 2**. which shows that there are differences between the two groups: mean ± SD (CV%) of GIR_max_ was 3.0308 ± 0.9512 mg/kg/min (31.39%) vs. 4.1939 ± 1.0278 mg/kg/min (24.51%); median of tGIR_max_ was 174.00 min (73.8~379.2 min) vs. 145.20 min (49.2~204 min); median of early tGIR_10%max_ was 11 min (3~30 min) vs. 15 min (2~23 min); median of early tGIR_50%max_ was 42 min (23~164 min) vs. 66 min (31~115 min). The relative bioavailability of GIR was calculated as follows:

**Table 2:** Summary of pharmacodynamic parameters for GIR (mg/kg/min) (pharmacodynamic parameter set).

Pharmacodynamic parameters (unit)	Group T	Group R
	(mean ± sd)	(mean ± sd)
GIR_max_(mg/kg/min)	3.0308 ± 0.9512	4.1939 ± 1.0278
AUC_GIR,0-2 h_(mg/kg)	158.542 ± 91.9319	224.819 ± 57.9694
AUC_GIR,0-4 h_(mg/kg)	422.510 ± 203.4403	614.073 ± 162.1420
AUC_GIR,0-8 h_(mg/kg)	956.126 ± 444.0072	1234.760 ± 443.3238
tGIR_max_(min)^a^	174.00 (73.8, 379.2)	145.20 (49.2, 204)
Onset of action		
early tGIR_10%, max_(min) ^a^	11 (3, 30)	15.0 (2, 23)
early tGIR_50%, max_(min) ^a^	42 (23, 164)	66 (31, 115)
F(%)	1.890 ± 1.0553	

^a:^ tGIR_max_ and onset of action are presented as median (minimum, maximum).

F=AUC_GIR,0-8 h, oral_/AUC_GIR,0-8 h, injection_* (Dose_injection_/Dose_oral_)*100%

The relative bioavailability of GIR in patients from Group T was calculated to be (1.890 ± 1.0553) %.

### Safety assessments

A total of 12 subjects were included in the safety analysis set. There was no occurrence of any adverse events during the study, including deaths, serious adverse events other significant adverse events, adverse events requiring special attention, and hypoglycemic events. No statistical significance was observed in laboratory parameters when compared with the baseline during this study.

## Discussion

This is the first clinical study of N11005, to evaluate its pharmacokinetics, pharmacodynamics, and safety in healthy subjects so as to verify its use as a prandial insulin.

Microemulsified human insulin (N11005) is a nanodroplet with a size smaller than 200 nm, which contributes to drug penetration in the gastrointestinal tract and absorption of protein drugs after oral administration. Its solid form overcomes the storage instability of human insulin in a liquid environment. The representation data of multiple batches of N11005 have demonstrated that this microemulsified drug is characterized by uniform size, good stability, and avoidance of a layered character in storage. Compared with insulin injection, oral insulin has the following dominant advantages. Firstly, it can relieve patients from discomfort due to frequent daily injections, hence higher compliance. Secondly, it can avoid the embarrassment caused by injection and is more convenient to administer on a daily basis because there are no restrictions regarding the administration environment. Thirdly, this oral insulin drug is cost friendly as it is easy to store and carry in granular form. There is no need for low-temperature storage by patients and for cold-chain transportation by manufacturers. Fourthly, it is more acceptable because its administration is so simple and convenient that patients can use it without training. They only need add water to emulsify the granule before administration. Fifthly, it can enter the liver through portal vein circulation after it is absorbed by the gastrointestinal tract (7, 8), which is identical to the normal physiology of insulin. In this way, it can reduce the risk of hyperinsulinemia, body weight gain caused by systemic insulin treatment, and hypoglycemia.

The rationale of dose selection for the study drug was based on the preclinical studies of N11005 in pharmacology and toxicology as well as the clinical trial dosing of other similar dosage forms. Considering the bioavailability of N11005 in dogs was 1.83%, 300 IU was determined as a dose of administration in the present study. During the study, the C-peptide levels and blood glucose levels of subjects reflected the stability of the euglycemic clamp platform. After oral administration of N11005 at a dose of 300 IU, C-peptide was remarkably inhibited, and GIRs also indicated excellent absorption of N11005.

For healthy people, beta cells in pancreatic islets will secrete insulin within 5 minutes after a meal due to stimulation of glucose, with an onset of action in 10-20 minutes, peak of action in 1-2 hours, and duration of action of 3-5 hours. This kind of rapid- and short-acting insulin is called prandial insulin whose major function is to normalize postprandial blood glucose (9, 10). According to the results of this study, the time to 10% of the maximal GIR (early tGIR_10%max_) was observed to be 11 minutes after oral insulin N11005 was administered at a dose of 300 IU to subjects, indicating the onset of action of the drug (11). In other clamp studies, the time until 50% of the maximal GIR (early tGIR_50%max_) was also used clinically to observe the onset of action of drugs. According to their study results, the early tGIR_50%max_ of conventional recombinant human insulins was about 1 hour (12–16), and rapid-acting insulins, 0.5~1 hour (12, 14–17). The early tGIR_50%max_ of oral insulin N11005 (300 IU) in this study was observed to be 42 minutes which was faster than that in the control group (66 minutes) and also within the range for rapid-acting insulins in literature. In spite of the fact that the duration of action of the study drug was difficult to evaluate exactly in this study, it was similar to that of the conventional insulin in the control group. To sum up, the study drug N11005 basically conforms to the characteristics of prandial insulin and its action profiles are close to the postprandial secretion pattern of regular human insulin. It can effectively control postprandial blood glucose in diabetic patients and help improve long-term blood glucose control.

The study drug is oral insulin most of which enters the liver through the portal vein after being absorbed in the digestive tract and forms a local high concentration in the liver with only a small amount joining systemic circulation. In this way, it inhibits the decomposition of glycogen in the liver and reduces the occurrence of peripheral hyperinsulinemia, which is identical to normal physiology. The fact that its relative pharmacokinetic bioavailability was lower than relative pharmacodynamic bioavailability might be due to the decrease of serum insulin concentration caused by insulin uptake and utilization by the liver and the first pass effect. In addition, no significant peak of serum insulin concentration was observed after administration in this study. Apart from the reasons above, it might also result from so high an insulin level in the circulation system that direct serum insulin in subjects became less obvious, and from great individual variation because of the small sample size in this study. Further observation will be conducted in the follow-up clinical trial. We did not use somatostatin to inhibit endogenous insulin and glucagon secretion and compared its hypoglycemic effect with oral insulin, which is a limitation of the study.

The adverse reactions of the study drug N11005 and the reference drug Novolin R were monitored during this study. No adverse events were discovered in any subjects.

## Conclusions

The present study preliminarily verified that N11005 exhibited prandial insulin characteristics in healthy Chinese male subjects. The relative bioavailability of GIR for a single dose of 300 IU oral insulin (N11005) administered in fasting condition was (1.890 ± 1.0553) % compared with subcutaneous injection of Novolin R. In general, reference and study drugs were both safe and well-tolerated.

## Data availability statement

The dataset supporting the conclusions of this article is included within the article.

## Ethics statement

The study was conducted at Beijing Hospital (the clinical study site) with the approval of its Drug Clinical Trial Institutional Ethics Committee in accordance with the Declaration of Helsinki.

## Author contributions

LG, QP, and XW conceived the experimental strategy, which was further developed by QZ, WL, XC, and YLZ. ZW researched data, contributed to the discussion, and WW provided statistical support. JH, W-HH, and Y-TY contributed to animal studies. YH, YJZ, and LL prepared the manuscript with input from all co-authors. All authors participated in the preparation and review of the manuscript. LG is the guarantor of this work and, as such, had full access to all of the data in the study and takes responsibility for the integrity of the data and the accuracy of the data analysis. All authors contributed to the article and approved the submitted version.
